# P14 Auditing the outcomes of the OPAT service of a London teaching hospital during the period of 2024

**DOI:** 10.1093/jacamr/dlaf239.018

**Published:** 2026-01-05

**Authors:** Adam Stafford, Hannah Kershaw, Winifred Magambo-Gasana, Annette Vanhein-Wallace, Rebecca Lester, Antonia Scobie, Natasha Karunaharan, Lucy Lamb, Stephen Mepham, Cordelia Coltart, Sophie Collier

**Affiliations:** Royal Free London NHS Foundation Trust, London, UK; Royal Free London NHS Foundation Trust, London, UK; Royal Free London NHS Foundation Trust, London, UK; Royal Free London NHS Foundation Trust, London, UK; Royal Free London NHS Foundation Trust, London, UK; Royal Free London NHS Foundation Trust, London, UK; Royal Free London NHS Foundation Trust, London, UK; Royal Free London NHS Foundation Trust, London, UK; Royal Free London NHS Foundation Trust, London, UK; Royal Free London NHS Foundation Trust, London, UK; Royal Free London NHS Foundation Trust, London, UK

## Abstract

**Objectives:**

We aimed to audit the Outpatient Parenteral Antimicrobial Therapy (OPAT) service at a London teaching hospital during 2024, in-line with the outcome monitoring set out in the *Updated good practice recommendations for OPAT in adults and children in the UK* (Chapman *et al*., 2019)

**Methods:**

We performed a retrospective review of electronic notes of all patients on the OPAT service during 2024 who had received either IV or complicated oral antimicrobials, defined as ‘oral regimens that require specific monitoring or associated with particular risk of toxicity’ (Chapmen *et al*., 2019). We extracted relevant clinical data to assess rates of readmission, treatment failure and adverse events.

**Results:**

Data from 162 patients was audited: 100 male and 62 female, with a median age of 62. 124 patients received IV antimicrobials, and 38 complicated oral antimicrobials – namely linezolid, co-trimoxazole and oral antifungals. The most common indications for IV antimicrobials were bronchiectasis (19%), pyelonephritis (12%) and liver abscesses (12%); with the most common indication for oral antimicrobials being diabetic foot infection (36%). The most commonly used IV antimicrobials were ertapenem (*n*=32), ceftriaxone (*n*=32) and ceftazidime (*n*=25). Of those on IV antimicrobials, 62% (*n*=77) were being treated with the aim of cure, 19% (*n*=19) improvement, and 8.9% (*n*=11) with palliative intent. In 87% (*n*=108) of these patients, the treatment aim was attained; this was without any complications in 74% (*n*=80). Of those who did not attain their treatment aim, the majority (57%) were in the palliative cohort. The most common complications were adverse drug reactions (11% of all patients) and line-related complications (6.5%). 16 patients (13%) were readmitted to hospital, all for a period of over 24 h. There was one case of *Clostridium difficile* infection, but no *Staphylococcus aureus* line-infections or death. Of those on complicated oral antimicrobials, 84% (*n*=32) were being treated with the aim of cure and 16% (*n*=6) with the aim of improvement. In 86% (*n*=33) of these patients, the treatment aim was attained; this was without complications in 66% (*n*=22). 11 patients (29%) suffered adverse drug reactions, but there were no other complications noted. No patients were readmitted to hospital.

**Conclusions:**

The heterogenous nature of published studies of OPAT data, combined with the difference in patient populations and collection methods makes direct comparisons difficult; nevertheless, the outcome data from our audit shows high rates of treatment success and low readmission and complication rates on par with other UK studies. Benchmarking against national data remains valuable in identifying areas for quality improvement, and our audit highlights the importance of multi-disciplinary team working for the successful running of an OPAT service.

